# High‐Power‐Density Rechargeable Hybrid Alkali/Acid Zn–Air Battery Performance Through Value‐Added Conversion Charging

**DOI:** 10.1002/advs.202402343

**Published:** 2024-04-04

**Authors:** Ximeng Yin, Wei Sun, Kai Chen, Zhiwen Lu, Junxiang Chen, Pingwei Cai, Zhenhai Wen

**Affiliations:** ^1^ Fujian Provincial Key Laboratory of Advanced Inorganic Oxygenated‐Materials College of Chemistry Fuzhou University Fuzhou Fujian 350108 China; ^2^ CAS Key Laboratory of Design and Assembly of Functional Nanostructures Fujian Provincial Key Laboratory of Nanomaterials State Key Laboratory of Structural Chemistry Fujian Institute of Research on the Structure of Matter Chinese Academy of Sciences Fuzhou 350002 P. R. China; ^3^ Fujian College University of Chinese Academy of Sciences Fuzhou 350002 P. R. China

**Keywords:** alkali/acid electrolytes, glycerol oxidation reaction, hybrid Zn–air battery, high power density, reduced charging voltage

## Abstract

Rechargeable Zn–air batteries (ZABs) are considered highly competitive technologies for meeting the energy demands of the next generation, whether for energy storage or portable power. However, their practical application is hindered by critical challenges such as low voltage, CO_2_ poisoning at the cathode, low power density, and poor charging efficiency Herein, a rechargeable hybrid alkali/acid Zn–air battery (h‐RZAB) that effectively separates the discharge process in an acidic environment from the charging process in an alkaline environment, utilizing oxygen reduction reaction (ORR) and glycerol oxidation reaction (GOR) respectively is reported. Compared to previously reported ZABs, this proof‐of‐concept device demonstrates impressive performance, exhibiting a high power density of 562.7 mW cm^−2^ and a high operating voltage during discharging. Moreover, the battery requires a significantly reduced charging voltage due to the concurrent utilization of biomass‐derived glycerol, resulting in practical and cost‐effective advantages. The decoupled system offers great flexibility for intermittently generated renewable power sources and presents cost advantages over traditional ZABs. As a result, this technology holds significant promise in opening avenues for the future development of renewable energy‐compatible electrochemical devices.

## Graphical abstract

A rechargeable hybrid Zn–air battery (h‐RZAB) is proposed by decoupling charging and discharging processes in separate alkaline and acidic electrolytes. which demonstrates a remarkable power density of 562.7 mW cm^−2^ and maintains a high operating voltage during discharging. Additionally, the battery requires a significantly reduced charging voltage due to the concurrent utilization of biomass‐derived glycerol.

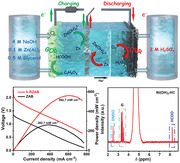

## 1 Introduction

Zn–air batteries (ZABs) have been regarded as a promising energy technology for renewable energy storage and portable power sources thanks to their multiple advantages, including high energy density, low cost, environmental friendliness, and superior safety features.^[^ ^1^ ^]^ They have attracted significant research attention in recent years with the aim of exploring low‐cost, high‐activity, and durable cathode catalysts for the oxygen reduction reaction (ORR) and oxygen evolution reaction (OER).^[^ ^2^ ^]^ Significant progress has been made in the development of precious‐metal‐free and high‐performance cathode catalysts for ZABs. However, there is still considerable scope for improving cathode catalysts by enhancing their activity and durability while simultaneously reducing costs.^[^ ^3^ ^]^ Additionally, the development of commercially competitive ZABs still faces at least two significant challenges. First, the poor reversibility between the oxygen evolution reaction (OER) and oxygen reduction reaction (ORR) often results in low efficiency during charging and discharging cycles, even when using benchmark precious‐metal catalysts such as platinum (Pt), iridium (Ir), or ruthenium (Ru) as electrode materials for O_2_‐involved electrocatalysis.^[^ ^3^, ^4^ ^]^ Second, the operating voltage of conventional ZABs is limited by the narrow voltage window of water solvent,^[^ ^5^ ^]^ significantly impeding their electrochemical performance. Furthermore, the presence of carbon dioxide (CO_2_) in the air can react with the alkaline electrolyte, leading to the formation of carbonate salt, which can cause degradation in the performance and stability of the Zn–air battery.

The hybrid Zn–air battery (h‐ZAB) has been proposed by coupling the Zn anode in alkali and the ORR cathode in acid to broaden the operating voltage window, which significantly enhances performance parameters such as voltage, power density, and energy density.^[^ ^6^ ^]^ Notably, the poisoning effect induced by the carbonation of CO_2_ in traditional ZAB can be mitigated because an acidic solution is used as a catholyte that prevents CO_2_ carbonation. However, charging with OER is not favorable in an acidic electrolyte, which presents critical issues in searching for low‐cost, durable, and high‐activity catalysts. In this context, charging in h‐ZAB is normally implemented by a mechanical route rather than an electrolytic route, which is yet highly desirable to cater to the intermittent renewably generated power.^[^ ^6d,e^ ^]^ Electro–oxidation of a molecule with a lower thermodynamic potential than OER has been proposed as the anodic reaction for charging or Zn electroplating.^[^ ^7^ ^]^ However, most electro–oxidation reactions are also thermodynamically and kinetically favorable in an alkaline solution.^[^ ^8^ ^]^

Taking into consideration the aforementioned points, we here propose a hybrid rechargeable Zn–air battery (h‐RZAB) that decouples the discharge–charge process in different electrolytes, pairing acidic ORR for discharging with alkaline glycerol oxidation reaction (GOR) for charging, respectively. The feasibility of this proof‐of‐concept hybrid device was demonstrated by developing precious metal‐free electrocatalysts for the ORR discharging reaction and the GOR charging reaction. The h‐RZAB can deliver a high power density among the reported Zn‐based batteries, and importantly, it can also upgrade glycerol during the charging process using a lower voltage than during discharging. This pioneering strategy represents a new approach to developing high‐efficiency rechargeable battery devices that synergize with chemical upgrading and renewable energy.

## 2 Results and Discussion

**Scheme 1:**
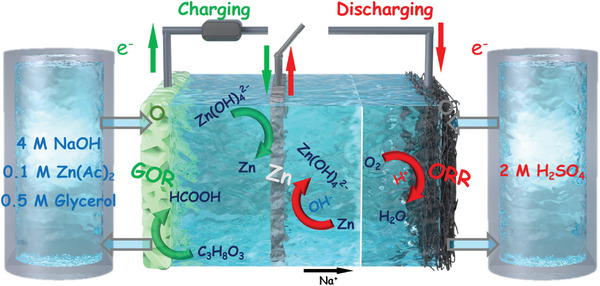
Schematic diagram of as‐proposed h‐RZAB.

Glycerol frequently emerges as a byproduct in the course of biodiesel production and various industrial procedures. The oxidation of glycerol presents a viable avenue for repurposing this residual substance, transforming it into valuable energy or compounds, thereby mitigating overall waste. The oxidation of glycerol can be seamlessly integrated into the biofuel production workflow. Through the conversion of glycerol into diverse compounds, it becomes an instrumental factor in the synthesis of biofuels, including but not limited to biodiesel and other sources of renewable energy. GOR manifests a lower oxidation potential compared to that of OER, rendering it a more energy‐conserving and efficient process. **Scheme** **1** illustrates the battery configuration and operation mechanism of the h‐RZAB, which consists of two chambers separated by a cation exchange membrane (CEM) containing flow acidic and alkaline electrolytes to prevent the chemical neutralization of alkali/acid. Flow electrolytes offer several advantages: 1) Flow electrolytes enable better mass transport of reactants to the electrode surfaces. The continuous flow of electrolytes helps maintain a consistent concentration of reactants, reducing concentration polarization and improving overall efficiency. 2) In continuous‐flow electrolytes, products of electrolysis can be continuously separated from the reaction mixture, facilitating downstream processing and product isolation. 3) Flow systems allow for flexibility in choosing electrolyte compositions. Different electrolytes can be tested and optimized for specific reactions, offering versatility in terms of the types of electrolysis processes that can be performed.

During discharging, the Zn plate in alkali serves as an anode and is oxidized to Zn(OH)_4_^2−^ with releasing electrons (Equation (1). The electrons then travel to the acidic cathode for ORR (Equation (2), while Na^+^ ions pass through the CEM from the anolyte to the catholyte to form a close circuit and maintain the neutrality of electrolytes under the forward bias.^[^ ^9^ ^]^ The h‐RZAB can theoretically liberate a voltage of 2.55 V thanks to its hybrid design with a pH gradient between anode and cathode that is capable of broadening the operating voltage window. During the charging process, Zn electroplating (Equation (4) is paired with electrocatalytic GOR (Equation (5) in the alkaline electrolyte. Notably, the charging voltage can be even much smaller than the discharging voltage in theory, thanks to the co‐contribution of electrochemical neutralization energy ^[^ ^10^ ^]^ and the substitution of OER with GOR during charging, which simultaneously offer additional profits of glycerol upgrading conversion.

Discharging:

Anode:

(1)

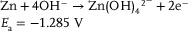




Cathode:

(2) $$\begin{array}{c} O_{2}+4H^{+}+4e^{-}\to 2H_{2}O\;\\ E_{c}=1.265\;V \end{array}$$

(3) $$V_{\mathrm{Discharging}}=\mathrm{Ec}-\mathrm{Ea}=2.55\;V$$

Charging:

Anode:

(4)

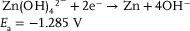




Cathode:

(5) $$\begin{array}{c} \;C_{3}H_{8}O_{3}+11OH^{-}\to 3\mathrm{CH}{O_{2}}^{-}+8H_{2}O+8e^{-}\;\\ E_{c}=-0.671\;V \end{array}$$

(6) $$V_{\mathrm{Charging}}=\mathrm{Ec}-\mathrm{Ea}=0.614\;\;V$$

**Figure 1:**
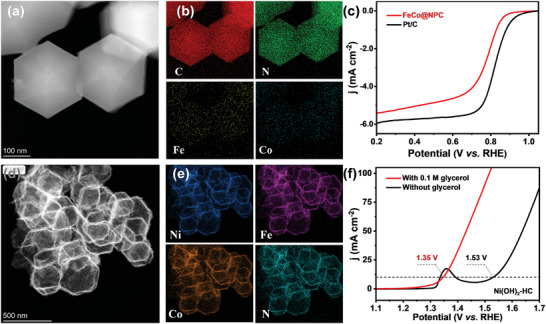
a,b) TEM image and corresponding element mapping of FeCo@PNC. c) LSV cures of FeCo@PNC and Pt/C in 0.1 m HClO_4_ solution. d,e) TEM image and corresponding element mapping of Ni(OH)_2_‐HC. f) LSV curves of Ni(OH)_2_‐HC in 1.0 m KOH solution with and without glycerol.

To operate the battery, N‐doped porous carbon with a trace of Fe and Co decorating nanohybrid (FeCo@NPC) was prepared as acidic electrocatalysts for ORR in the h‐RZAB, while Ni(OH)_2_ hollow cage (HC) was fabricated as anodic electrocatalysts for GOR upon charging to pair with cathodic Zn electroplating,^[^ ^11^ ^]^ respectively. Details on the synthetic processes and the characteristics of the associated samples are provided in the supporting information. (Figures S1–S4, Supporting Information). Scanning electron microscope (SEM) images show that the FeCo@NPC is standard rhombic dodecahedrons with uniform size (≈200 nm) and smooth surface (Figure S2, Supporting Information), indicating no change in the morphology and structure of ZIF is observed confirmed by X‐ray diffraction (XRD) pattern. Transmission electron microscope (TEM) images show that FeCo@NPC maintains the dodecahedron structure of the ZIF‐8 precursor and does not contain observable nanoparticles (**Figure** **1**a). The elemental mapping of FeCo@NPC reveals that Fe, Co, C, and N are uniformly distributed throughout the sample (Figure 1b). The poorly defined diffraction ring in Figure S2j (Supporting Information) confirms that the carbon in FeCo@NPC has an amorphous structure, which is consistent with the result of the XRD pattern shown in Figure S2f (Supporting Information). X‐ray photoelectron spectrum (XPS, Figure S3, Supporting Information) results prove the existence of N and C elements. The weak XPS signals for Fe and Co elements are primarily due to their low content^[^ ^12^ ^]^ and the thick carbon layer coating (Figure S2i, Supporting Information), as evidenced by weak element mapping signals for Fe and Co (Figure 1b). The Raman spectra indicate that FeCo@NPC has a higher density of defects (*I*_D_/*I*_G_ = 1.09, Figure S4, Supporting Information), which may enhance the ORR activity.

The electrocatalytic properties of FeCo@NC for ORR were evaluated by linear sweep voltammetry (LSV) on the rotating disk electrode (RDE) in an O_2_‐saturated 0.1 m HClO_4_ solution. As shown in Figure 1c, FeCo@NPC exhibits a half‐wave potential (*E*_1/2_) of 0.78 V, this value is comparable to that (0.82 V) of the commercial Pt/C (20 wt.%). In addition, both FeCo@NPC and Pt/C show similar slope values of Tafel plots with 77.6 and 77.1 mV dec^−1^ (Figure S5, Supporting Information), respectively, indicating the former has a comparable kinetics to Pt/C toward electrocatalysis of ORR. The electrochemical impedance spectroscopy (EIS) also verifies the FeCo@NPC shows a comparable charge transfer rate to the Pt/C upon electrocatalysis of ORR (Figure S6, Supporting Information). Based on the Kouteckey–Levich (K–L) plots, the electrocatalysis of FeCo@NPC for ORR majorly proceeds in a four‐electron pathway in the range of 0.4–0.75 V (Figure S7, Supporting Information), which is well in accordance with rotation ring disk electrode (RRDE) result (Figure S8, Supporting Information) that reveals the H_2_O_2_ yield of FeCo@NPC is less than 3%. Notably, the FeCo@NPC displays more durable stability than the Pt/C, as evidenced by the higher current retention rate of FeCo@NPC (Figure S9, Supporting Information).

To enable charging in h‐RZAB, Ni(OH)_2_ hollow cage, *i. e*., Ni(OH)_2_‐HC, was developed as anodic electrocatalysts for glycerol oxidation reaction (GOR). Ni(OH)_2_‐HC was characterized using a variety of techniques, such as XRD, SEM, TEM, and XPS (Figures S10–S12, Supporting Information), demonstrating the catalysts are Ni(OH)_2_ in phase. A typical TEM image of Ni(OH)_2_‐HC displaying a hollow octahedral structure is shown in Figure 1d, and the elements of Ni, Fe, and Co are uniformly distributed throughout the octahedral framework (Figure 1e), while the signals for Fe and Co are contributed to the residue of the precursor. The distinct signals of Fe and Co in the elemental mapping can be attributed to the hollow porous structure, facilitating easier exposure. For electrocatalytic testing, the Nickel foam served as a current collector loading Ni(OH)_2_‐HC. Figure 1f compares the LSV curves of Ni(OH)_2_‐HC in 1.0 m KOH in the presence and absence of 0.1 m glycerol. When 0.1 m G was introduced into the electrolyte, the current density increased significantly, and the onset potential decreased to 1.24 V compared to OER. Moreover, the Ni(OH)_2_‐HC presents a current density of 10 mA cm^−2^ at an applied voltage of 1.35 V in the electrolyte with 0.1 m glycerol. This value significantly shifts positively relative to OER in the solution without glycerol. Notably, the Ni(OH)_2_‐HC also shows accelerated reaction kinetics toward GOR relative to OER, as manifested by a smaller Tafel slope and a lower charge transfer resistance (Figure S13, Supporting Information). The lower charge transfer resistance observed in the GOR compared to the OER can be ascribed to various factors. First, GOR typically entails a reaction with a lower oxidation potential in contrast to OER. Reactions with lower potential generally exhibit swifter electron transfer kinetics, thereby resulting in a diminished charge transfer resistance. Second, the specific reaction kinetics of GOR may favor a more facile electron transfer process, contributing to a lower charge transfer resistance. This phenomenon may be influenced by factors such as the nature of reactants, reaction pathways, and intermediates involved. Lastly, the catalysts employed in GOR and OER can possess distinct properties, including their capacity to facilitate electron transfer. If the catalyst utilized for GOR is more efficacious in promoting electron transfer, it is likely to contribute to a diminished charge transfer resistance. The potentiostatic method was further employed to evaluate the stability of the catalyst. The imperceptible decrease in current density over 12 h of continuous electrolysis (Figure S14, Supporting Information) indicates the high stability of Ni(OH)_2_‐HC.

**Figure 2:**
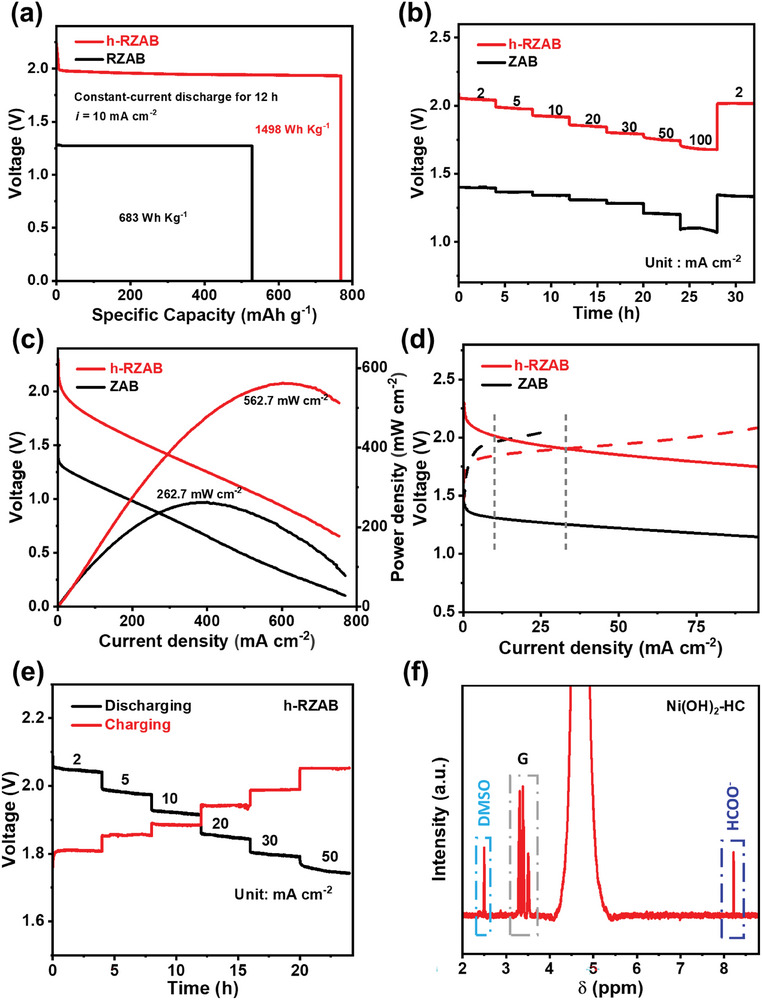
a,b) Electrochemical performance of h‐ZAB. a) Voltage versus specific capacity curves, from which we can calculate the energy density; b) Discharging voltages at different current densities; c) Discharging polarization curves and corresponding power density curves; d) Charging and discharging polarization curves of h‐RAZAB and ZAB. e) Charging and discharging voltages of h‐RAZAB at different current densities. f) ^1^H NMR measurements of glycerol oxidation in the charging process of h‐ZAB.

Based on the desirable activity of the two electrocatalysts, we set up a h‐RZAB by utilizing FeCo@NPC as the cathode catalyst for discharging and Ni(OH)_2_‐HC as the anode electrocatalyst for charging (Figure S15, Supporting Information). For comparison, we assembled a conventional ZAB with a Zn anode, a FeCo@NPC cathode, and an electrolyte of 4.0 m NaOH solution. The h‐RZAB exhibits remarkable advantages over the traditional ZAB. First, it can liberate an open‐circuit voltage (*V*_OC_) of 2.23 V, as measured by a multimeter (Figure S15, Supporting Information). This value is significantly higher than the typical *V*_OC_ of ≈1.5 V for the traditional ZAB. Even when operating at a current density of 10 mA cm^−2^, the h‐RZAB can supply a voltage of as high as 2.01 V (**Figure** **2**a), which is 0.7 V higher than that of the conventional ZAB (1.31 V). Second, the corresponding energy density of h‐RZAB is 1498 Wh ${\mathrm{kg}}_{\mathrm{Zn}}^{-1}$ calculated by the integral area of voltage versus capacity curves, also higher than that of ZAB (683 Wh ${\mathrm{kg}}_{\mathrm{Zn}}^{-1}$, Figure 2a). Additionally, the h‐RZAB demonstrates fast rate performance, achieving a current density of 100 mA cm^−2^ at an operating voltage of 1.69 V. (Figure 2b), which is even higher than the theoretical voltage of traditional ZAB (≈1.65 V). Moreover, the h‐RZAB can release a considerably large current density even at a high operating voltage with a maximum power density of 562.7 mW cm^−2^ (Figure 2c), this value notably presents the highest power density among all the ZAB reported so far to the best of our knowledge,^[^ ^3^, ^13^ ^]^ and is above twice higher than that of ZAB (262.7 mW cm^−2^). Lastly, compared with the traditional ZAB, the h‐RZAB can deliver a higher voltage (≈2.0 V) during discharging but requires a lower voltage (≈1.5 V) upon charging (Figure 2d). One can observe that during charging, the h‐RZAB with Ni(OH)_2_‐HC as anode electrocatalyst for GOR only requires an applied voltage of 1.84 V at 10 mA cm^−2^ (Figure 2d), which is even lower than that of ZAB with the state‐of‐the‐art Pt/C and/or RuO_2_ cathode (1.96 V).

Interestingly, the required charging voltage for h‐RZAB is lower than the discharging voltage at low current densities. While the voltage gap between charging and discharging can reach as high as 0.71 V for ZAB at 10 mA cm^−2^. Figure 2e depicts the charging/discharging profiles of h‐RZAB at various current densities, showing that the discharging voltages remain higher than the charging voltages until the current density exceeds 10 mA cm^−2^. Additionally, the h‐RZAB demonstrates lower charging voltages at different current densities without the effect of bubbles and long‐term charging stability compared to conventional ZAB (Figure S16, Supporting Information). These findings suggest that the hybrid alkali/acid‐electrolyte design can significantly enhance battery performance due to the co‐contribution of electrochemical neutralization energy and broadened voltage window.^[^ ^14^ ^]^ Furthermore, ^1^H Nuclear Magnetic Resonance (NMR) measurement is carried out to identify the product of GOR during the charging process of h‐RZAB. As displayed in Figure 2f, the peaks located at around 8.3 ppm are derived from the signals of formate,^[^ ^15^ ^]^ and the Faradic efficiency of formate is estimated to be ≈85%, implying biomass updating during the charging process of h‐RZAB.

To optimize the performance of h‐ZAB, we established two types of h‐ZABs, namely h‐RZAB‐I and h‐RZAB‐II, for comparison. In h‐RZAB‐I, ORR occurs in acid during discharging, while OER takes place in alkali during charging (Figure S17a, Supporting Information). In h‐RZAB‐II, both ORR and OER take place in the acidic electrolyte during the charging and discharging process (Figure S17b, Supporting Information). It is noteworthy that h‐RZAB‐I and h‐RZAB‐II require higher charging voltage compared to the aforementioned h‐RZAB (Figure S17c, Supporting Information). The charging mode has a significant effect on the required voltage, with h‐RZAB needing a higher voltage than h‐RZAB‐I and h‐RZAB‐II, which can be attributed to the variation of the potential of the three charging reactions in the order: *E*_GOR in alkali_ < *E*_OER in alkali_ < *E*_OER in acid_. The stability of the proposed h‐RZAB was assessed through a cycle involving 5 h of discharging followed by 5 h of charging. It is demonstrated that the h‐RZAB can maintain considerable long‐term stability within 300 h (Figure S18, Supporting Information). Despite the slight increase in charging voltage and decrease in discharging voltage observed during cycling, these effects can be mitigated by refreshing the electrolytes and replacing the Zn anode.

**Figure 3:**
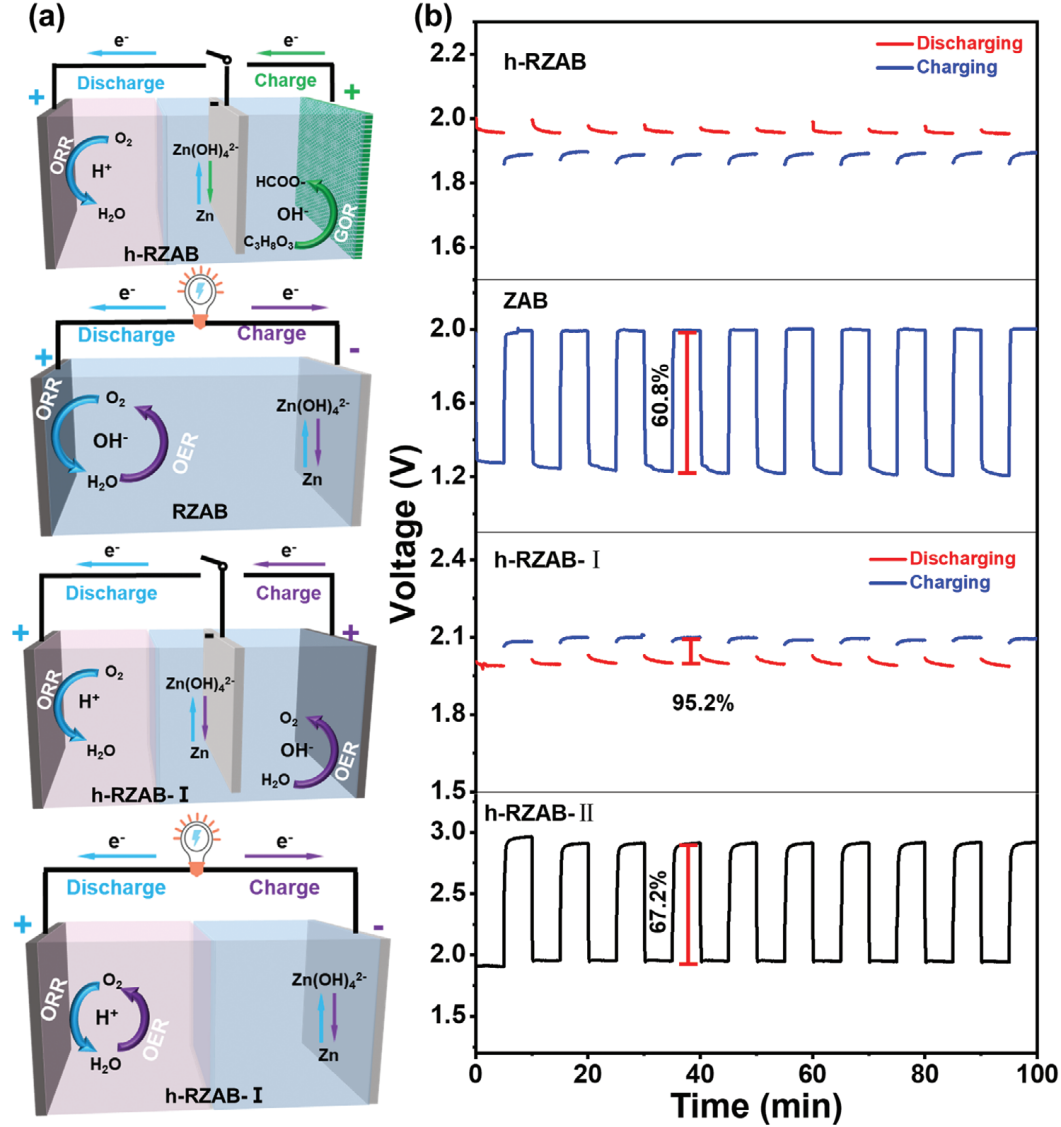
a) Schematic graphs and b) Galvanostatic charging/discharging profiles for h‐RZAB, ZAB, h‐RZAB‐I, and h‐RZAB‐II, respectively.

Round‐trip energy efficiency is a crucial factor in evaluating the performance of rechargeable batteries, which can be expressed as the ratio of the electrical energy output during discharge to input during charging. The round‐trip efficiency is influenced by various factors, including the design of the battery, materials used, and operating conditions. Achieving high round‐trip efficiency is significant for the economic viability and overall effectiveness of energy storage systems. In our study, we assessed this efficiency using the galvanostatic charging and discharging (GCD) technique, as shown in **Figure** **3**. The h‐RZAB exhibited a round‐trip efficiency above 100%, as its charging voltage (1.89 V) was lower than its discharging voltage (1.96 V). This indicates that the decoupled h‐RZAB significantly enhances the utilization efficiency of electrons, while also upgrading chemicals. On the other hand, the h‐RZAB‐I and h‐RZAB‐II deliver similar discharging voltages to h‐RZAB but much higher charging voltages, resulting in lower round‐trip efficiencies (95.2% for h‐RZAB‐I and 67.2% for h‐RZAB‐II, respectively). While ZAB displays lower discharging voltage and higher charging voltage in comparison to h‐RZAB, leading to a much lower round‐trip efficiency of 60.8%. These results further demonstrate that the hybrid alkali/acid electrolyte can remarkably improve the discharging performance of h‐RZAB, while the decoupling of charging by virtue of electrochemical glycerol oxidation can lower the charging voltage of h‐RZAB, jointly contributing to the high round‐trip efficiency. Moreover, during the charging process, the formate can be produced during GOR, achieving the conversion of value‐added products.

## 3 Conclusion

In conclusion, we have demonstrated a hybrid rechargeable Zn–air battery (h‐RZAB) that decouples the charging and discharging process using an acidic oxygen reduction reaction (ORR) discharging cathode and an alkaline glycerol oxidation reaction (GOR) charging anode. The h‐RZAB offers significant enhancements to battery electrochemical performance, including a high operating voltage, a maximum power density, and a high round‐trip efficiency of above 100% at a current density of 10 mA cm^−2^. These remarkable features, such as high voltage, high energy efficiency, and value‐added product generation, position the h‐RZAB as a promising candidate for electrochemical systems and provide a way to store and harvest intermittent renewable energy effectively and flexibly.

## Conflict of Interest

The authors declare no conflict of interest.

## Author Contributions

X.Y. and W.S. contributed equally to this work. X.Y., W.S., K.C., P.C., and Z.W. performed conceptualization; X.Y., W.S. wrote the original draft; X.Y., W.S., Z.L., P.C., and Z.W. wrote, reviewed and edited the final manuscript; X.Y., W.S. J. C. performed formal analysis; P.C. and Z.W. supervised all aspects of the work.

## Supporting information

Supporting Information

## Data Availability

The data that support the findings of this study are available from the corresponding author upon reasonable request.

## References

[1] a) W. Sun , F. Wang , B. Zhang , M. Zhang , V. Küpers , X. Ji , C. Theile , P. Bieker , K. Xu , C. Wang , M. Winter , Science 2021, 371, 46; 33384369 10.1126/science.abb9554

[2] a) M. Luo , Z. Zhao , Y. Zhang , Y. Sun , Y. Xing , F. Lv , Y. Yang , X. Zhang , S. Hwang , Y. Qin , J.‐Y. Ma , F. Lin , D. Su , G. Lu , S. Guo , Nature 2019, 574, 81; 31554968 10.1038/s41586-019-1603-7

[3] a) S. S. Shinde , J. Y. Jung , N. K. Wagh , C. H. Lee , D.‐H. Kim , S.‐H. Kim , S. U. Lee , J.‐H. Lee , Nat. Energy 2021, 6, 592;

[4] a) Z. Pei , Y. Huang , Z. Tang , L. Ma , Z. Liu , Q. Xue , Z. Wang , H. Li , Y. Chen , C. Zhi , Energy Storage Mater. 2019, 20, 234;

[5] a) L. Suo , O. Borodin , Y. Wang , X. Rong , W. Sun , X. Fan , S. Xu , M. A. Schroeder , A. V. Cresce , F. Wang , C. Yang , Y.‐S. Hu , K. Xu , C. Wang , Adv. Energy Mater. 2017, 7, 1701189;

[6] a) X. Yu , A. Manthiram , Joule 2017, 1, 453;

[7] a) G. Wang , J. Chen , P. Cai , J. Jia , Z. Wen , J. Mater. Chem. A 2018, 6, 17763;

[8] H. Jiang , J. Xia , L. Jiao , X. Meng , P. Wang , C.‐S. Lee , W. Zhang , Appl. Catal. B 2022, 310, 121352.

[9] M. Park , E. S. Beh , E. M. Fell , Y. Jing , E. F. Kerr , D. Porcellinis , M. A. Goulet , J. Ryu , A. A. Wong , R. G. Gordon , J. Cho , M. J. Aziz , Adv. Energy Mater. 2019, 9, 1900694.

[10] a) P. Cai , W. Sun , J. Chen , K. Chen , Z. Lu , Z. Wen , Adv. Energy Mater. 2023, 13, 2301279;

[11] a) C. Guan , X. Liu , W. Ren , X. Li , C. Cheng , J. Wang , Adv. Energy Mater. 2017, 7, 1602391;

[12] M. Xiao , J. Zhu , L. Ma , Z. Jin , J. Ge , X. Deng , Y. Hou , Q. He , J. Li , Q. Jia , S. Mukerjee , R. Yang , Z. Jiang , D. Su , C. Liu , W. Xing , ACS Catal. 2018, 8, 2824.

[13] a) T. Tang , W. J. Jiang , X. Z. Liu , J. Deng , S. Niu , B. Wang , S. F. Jin , Q. Zhang , L. Gu , J. S. Hu , L. J. Wan , J. Am. Chem. Soc. 2020, 142, 7116; 32196325 10.1021/jacs.0c01349

[14] a) C. Lin , S.‐H. Kim , Q. Xu , D.‐H. Kim , G. Ali , S. S. Shinde , S. Yang , Y. Yang , X. Li , Z. Jiang , J.‐H. Lee , Matter 2021, 4, 1287;

[15] a) L. Fan , Y. Ji , G. Wang , J. Chen , K. Chen , X. Liu , Z. Wen , J. Am. Chem. Soc. 2022, 144, 7224; 35404594 10.1021/jacs.1c13740

