# Supplementary material for: High‐Power‐Density Rechargeable Hybrid Alkali/Acid Zn–Air Battery Performance Through Value‐Added Conversion Charging
Source: Adv Sci (Weinh). 2024 Apr 4;11(23):2402343. doi: 10.1002/advs.202402343 (PMC11187864; doi:10.1002/advs.202402343)
Supplement: Supplementary file 1 — Supporting Information [file ADVS-11-2402343-s001.pdf]

ADVANCED SCIENCE
Open Access

Supporting Information

for *Adv. Sci.*, DOI 10.1002/advs.202402343

High-Power-Density Rechargeable Hybrid Alkali/Acid Zn–Air Battery Performance Through Value-Added Conversion Charging

Ximeng Yin, Wei Sun, Kai Chen, Zhiwen Lu, Junxiang Chen, Pingwei Cai and Zhenhai Wen**

Supporting Information for

High-Power-Density Rechargeable Hybrid Alkali/Acid Zn-Air Battery Performance through Value-Added Conversion Charging

Ximeng Yin,[1, 2+] Wei Sun,[1, 2+] Kai Chen,[2, 3] Zhiwen Lu,[2, 3] Junxiang Chen,[2, 3] Pingwei Cai, [1, 2, *] Zhenhai Wen[2, 3, *]

[1] Fujian Provincial Key Laboratory of Advanced Inorganic Oxygenated-Materials, College of Chemistry, Fuzhou University, Fuzhou, Fujian 350108, China

[2] CAS Key Laboratory of Design and Assembly of Functional Nanostructures, Fujian Provincial Key Laboratory of Nanomaterials, State Key Laboratory of Structural Chemistry, Fujian Institute of Research on the Structure of Matter, Chinese Academy of Sciences, Fuzhou 350002, P. R. China

*Correspondence: Cai2022@fzu.edu.cn, Wen@fjirsm.ac.cn

[3] Fujian College, University of Chinese Academy of Sciences Fuzhou, Fuzhou 350002, P. R. China

[+] These authors contributed equally to this work.

Experimental section

Materials

Cobalt (II) nitrate hexahydrate ($Co(NO_3)_2·6H_2O$), 2-methylimidazole (2-MI, $C_4H_6N_2$), Polyethylene-polypropylene glycol (P123) with a number-average molecular weight (M_w) = 5800 g/mol, and ruthenium (IV) oxide (RuO_2) were purchased from Shanghai Macklin Biochemical Co., Ltd. Zinc (II) Nitrate Hexahydrate ($Zn(NO_3)_2·6H_2O$), sodium hydroxide (NaOH) were purchased in Sinopharm Chemical Reagent Co., Ltd. Nafion D-521 dispersion (5% w/w in water and 1-propanol, ≥ 0.92 meq/g exchange capacity) was purchased from Alfa Aesar. Iron(III) acetylacetonate

($C_{15}H_{21}FeO_6$) and nickel (II) chloride hexahydrate ($NiCl_2·6H_2O$) were from Shanghai Aladdin Biochemical Technology Co. Ltd. Pt/C (20 wt%) and carbon paper (HCP120) were purchased from Shanghai Hesen Electrical Co., Ltd. All reagents were analytical grade and used as received without further purification.

Preparation of FeCo@NPC for oxygen reduction reaction.

The FeCo@NPC materials were obtained through a one-step pyrolysis method, involving the calcining the precursor ZIF@P123. Firstly, both solutions, solution A and solution B, were prepared. Solution A was prepared by dissolving 6.5 g of 2-MI and 0.353 g of $C_{15}H_{21}FeO_6$ in 80 mL of methanol. Solution B was prepared by dissolving 3.0 g $Zn(NO_3)_2·6H_2O$, 0.021 g $Co(NO_3)_2·6H_2O$, and 0.4 g of P123 in 40 mL of methanol and 10 ml of deionized (DI) water. After stirring the two solutions until they were clear, the resulting solution, *i.e.*, solution C, was further sonicated for half an hour before solution B was quickly added to solution A while stirring to obtain solution C. Solution C was stirred for 24 h prior to centrifugation. The precursor ZIF@P123 was obtained by centrifugation at 10,000 rpm for 5 min, washed three times with methanol, and dried at 60°C overnight in a vacuum drying box. After grinding, the precursor was placed into a porcelain boat and heated up to 900°C at a rate of 5°C min^{-1} under Ar/H_2 (10%) atmosphere. The catalytic materials were obtained after natural cooling.

Preparation of $Ni(OH)_2$/HC for glycerol oxidation reaction.

To prepare the hollow ZIF material modified with $Ni(OH)_2$ on the surface, named $Ni(OH)_2$/HC, 100 mg of the ZIF@P123 precursor and 380 mg of $NiCl_2·4H_2O$ were dissolved in 20 mL and 40 mL of methanol solution, respectively. The latter solution was poured into the former to obtain a mixed solution, which was stirred for 30 minutes. The resulting liquid was transferred to a 50 mL reactor and kept in an oven at 80°C for 2 h. After the reaction, the solution was centrifuged at 8000 rpm for 5 minutes to collect the precipitate, which was washed with methanol and freeze-dried to obtain the desired $Ni(OH)_2$/HC material.

Characterization: The morphology of the samples was investigated by scanning electron microscopy (SEM, Zeiss-Sigma 300) and transmission emission electron microscopy (TEM, FEI F20). X-ray powder diffraction (XRD) patterns were obtained using an X-ray diffractometer (Miniflex 600, Rigaku) at 40 kV and 15 mA using Cu-Kα radiation (λ = 1.54178 Å). Raman spectra were obtained on a LabRAM HR with a 532 nm excitation laser. X-ray photoelectron spectra (XPS) were taken on ESCALAB 250Xi (Thermo Scientific) XPS spectrometer with an Al Kα as the excitation source (1486.6 eV) to analyze the elemental chemical composition. Specific surface area and pore size distribution of the samples was examined by the Brunauer-Emmett-Teller (BET) method using nitrogen adsorption and desorption isotherms on a Micromeritics Instrument Corporation sorption analyzer (Micromeritics TriStar II 3020).

Electrochemical measurements: All of the electrochemical measurements were carried out in a three-electrode system using a CHI 760E electrochemical workstation (Shanghai Chenhua, China), in which carbon rod was served as the counter electrode and Ag/AgCl (saturated potassium chloride) and Hg/HgO (1.0 M KOH) as reference electrodes. The measured potentials were converted into the reversible hydrogen electrode (RHE) according to the Nernst equation ($E_{RHE} = E_{measured} + 0.0591 \times pH + E_{reference}$). To prepare the working electrode ink, 5.0 mg of grinding catalyst was dispersed in 1000 μL of a mixed solution of ethanol and DI water with a volume ratio of 1:1 containing 50 μL of 5% Nafion solution, which utilized ultrasonic treatment for 30 minutes to form a homogeneous ink. For comparison, 20 wt% Pt/C with the same mass loading was prepared.

For the oxygen reduction reaction (ORR) experiment, 0.1 M KOH and 0.1 M $HClO_4$ aqueous solutions were used as the test electrolyte, O_2 was continuously bubbled during the test and supplied for 30 minutes before the test to ensure oxygen saturation. The rotating ring disk electrode (RRDE, 5.0 mm in diameter) was used as the working electrode. 8 μL of ink was dropped while rotating ring disk electrode (RRDE, 4.0 mm in diameter), the working electrode, rotated at 400 rpm, on which the mass loading of active materials was about 0.5 mg cm^{-2}. Cyclic voltammetry (CV) test was performed

at a scan rate of 20mV s^{-1}. Linear sweep voltammetry (LSV) was performed with a sweep rate of 5 mV s^{-1} at different speeds (400, 625, 900, 1225, 1600, 2025, 2500 rpm). For comparison, the same operation was performed in Ar, except that LSV test was only performed at 1600 rpm. The ORR stability test was performed at a constant potential of 0.8 V with a rotating speed of 1600 rpm. The electron transfer number (n) could be calculated from the K-L equations (1) and (2):

$$\frac{1}{j} = \frac{1}{j_L} + \frac{1}{j_K} = \frac{1}{B\omega^{1/2}} + \frac{1}{j_K} \quad (1)$$

$$B = 0.62nFC_{O_2}D_{O_2}^{2/3}V^{-1/6} \quad (2)$$

where j is the measured current density, j_K and j_L are the kinetic and limiting current densities, respectively, ω is the electrode rotation speed; F is the Faradaic constant (96,485 C mol^{-1}), C_{O_2} is the O_2 concentration (solubility) in electrolyte (1.2 × 10^{-6} mol cm^{-3}), D_{O_2} is the O_2 diffusion coefficient in electrolyte (1.90 × 10^{-5} cm^2 s^{-1}), and V is the kinematic viscosity of the electrolyte solution (0.01 cm^2 s^{-1}).

For the RRDE measurements, a ring potential of 1.4 V was set, the percentage of peroxide species ($y_{peroxide}$) relative to the total products and the electron transfer number (n) can be calculated by the following equations:

$$n = \frac{4NI_d}{NI_d + I_r} \quad (3)$$

$$y_{peroxide} = \frac{200I_r}{NI_d + I_r} \quad (4)$$

where N is the current collection efficiency of RRDE (N=0.4 in this work), I_r is the ring current, and I_d is the disk current.

The oxygen evolution reaction (OER) and glycerol oxidation reaction (GOR) tests were to drop 100 μL ink on 1 × 1 cm^2 nickel foam (NF) as working electrode for LSV test at a scan rate of 5 mV s^{-1} in 1.0 M KOH solution saturated with Ar, setting IR compensation to 90% and the latter test solution contained 0.1 M glycerol. The CV curves were measured at scan rates of 5, 10, 15, 20, 25, 30, 35 and 40 mV s^{-1}, separately. Because a specific capacitance of 1 cm^2 flat surface-area (C_s) generally corresponds to 40 mF cm^{-2}, the calculation formula of ECSA was as follows:

$$ECSA = \frac{C_{dl}\ of\ catalyst}{0.04} \quad (5)$$

The electrochemical impedance spectroscopy (EIS) measurements for ORR, OER, and GOR were carried out from 100 kHz to 100 mHz at the voltage of 0.8 V and 1.4 V, respectively.

Zinc-air battery (ZAB) test

The traditional alkaline zinc-air battery was assembled, in which carbon paper loaded 1.5 mg of FeCo@NPC catalyst with an area of 1.0 cm^2 as the air cathode, polished zinc flakes as the anode, and 6 M KOH as the electrolyte. For comparison, 1.5 mg of 20 wt% Pt/C was used to prepare air electrode. The discharge LSV curve of ZAB was tested on the CHI 760E electrochemical workstation. Continuously discharged with different currents (2, 5, 10, 20, 30, 2 mA) on the LAND test system (CT3001A, Wuhan LAND Electronic Co. Ltd, Wuhan, China) to monitor the battery voltage and obtained the battery's rate performance. Discharged at a constant current of 10 mA for 12 h, weighed the mass of the zinc flakes before and after the test, and calculated the battery capacity as follow:

$$C = \frac{Q}{\Delta m} = \frac{It}{\Delta m} \tag{7}$$

Rechargeable zinc air battery (RZAB) test

For comparison, an alkaline rechargeable zinc-air battery was assembled. Carbon paper loaded with a 1.5 mg mixture of 20 wt% Pt/C and RuO_2 powder with a mass ratio of 1:1 was used as the air electrode, in which polished zinc foil as the anode, and 6.0 M KOH containing 0.2 M $Zn(Ac)_2$ as the electrolyte. Used LSV technology on CHI 760E electrochemical workstation to obtain the polarization curve of charging and discharging. The rest of the tests were carried out on the LAND test system, such as rate performance, capacity calculation, and constant current charge and discharge cycles.

Acid-base asymmetric rechargeable zinc-air battery-I (ARZAB-I)

The carbon paper loaded with 1.5 mg of Pt/C and RuO_2 with a mass ratio of 1:1 was the air cathode reacting under the acidic electrolyte environment of 2.0 M H_2SO_4. And the polished zinc flake served as the anode at 4.0 M NaOH + 0.1 M $Zn(Ac)_2$ in the alkaline electrolyte environment.

As above, performed the charge and discharge LSV test on the electrochemical workstation and the constant current charge and discharge cycle test on the LAND test system.

Acid-base asymmetric rechargeable zinc-air battery-II (ARZAB-II)

The 1.5 mg Pt/C catalyst loaded on carbon paper was the cathode during discharge, in the acidic environment of 2.0 M H_2SO_4. The polished zinc flakes as the negative electrode and 1.5 mg RuO_2 loaded on the NF as the anode during charging in the alkaline environment of 4.0 M NaOH including 0.1 M $Zn(Ac)_2$. The charge and discharge test was carried out on the electrochemical workstation, in which Pt/C and Zn were subjected to discharge test, and RuO_2 and Zn were subjected to charge reaction. As above, carried out the rate, capacity and constant current charge and discharge cycle test on the LAND test system.

Glycerol oxidation charging-acid-base asymmetric rechargeable zinc-air battery (h-RZAB)

Carbon paper supported 1.5 mg FeCo@NPC catalyst acted as the ORR catalyst in the 2.0 M H_2SO_4 acidic environment, as the air cathode. The nickel foam supported 1.0 mg $Ni(OH)_2$/HC acted as the catalyst for GOR in the alkaline environment of 4.0 M NaOH containing 0.1 M $Zn(Ac)_2$ and 0.5 M glycerol. Performed the corresponding test on the workstation as above.

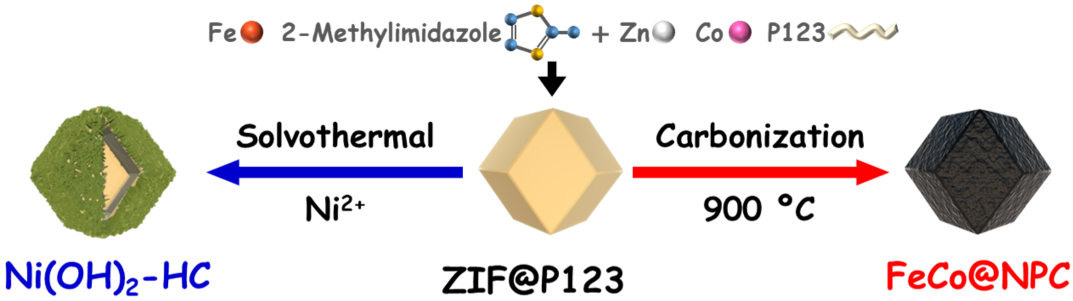

Figure S1. Schematic diagram of the synthesize of FeCo@NPC and $Ni(OH)_2$-HC.

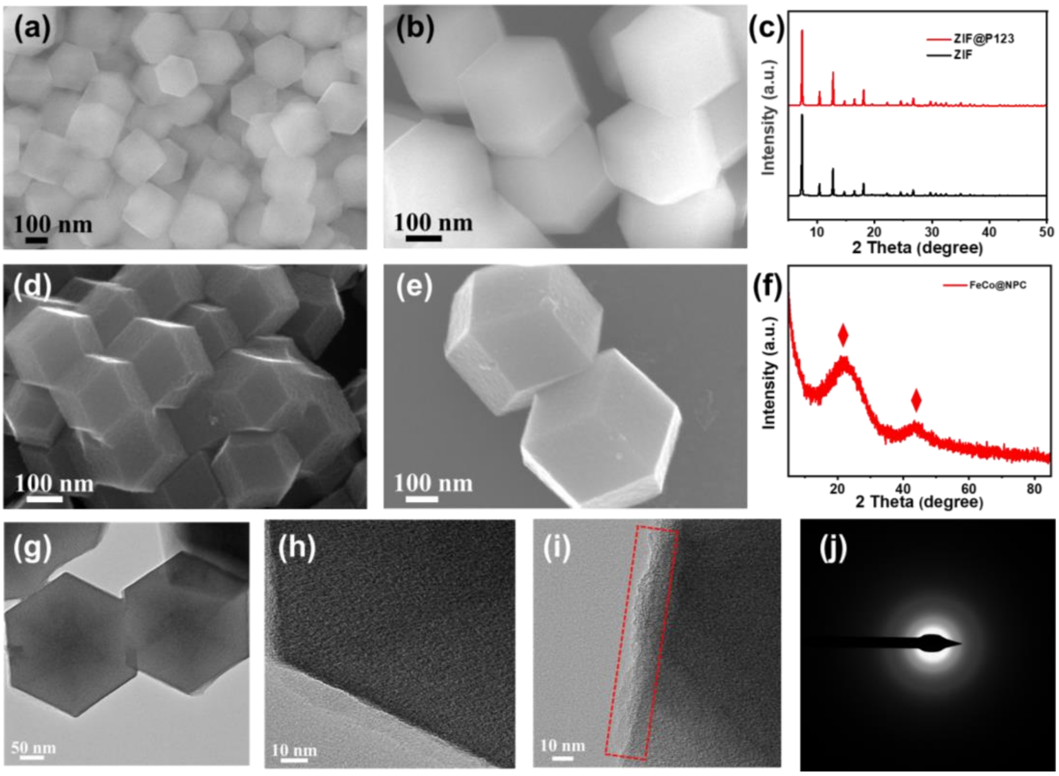

Figure S2. (a, b) SEM images of ZIF@P123. (c) XRD patterns of ZIF and ZIF@P123. (d, e) SEM images of FeCo@NPC. (f) XRD patterns of FeCo@NPC. (g-i) TEM images of FeCo@NPC. (j) Selected area electron diffraction pattern of FeCo@NPC.

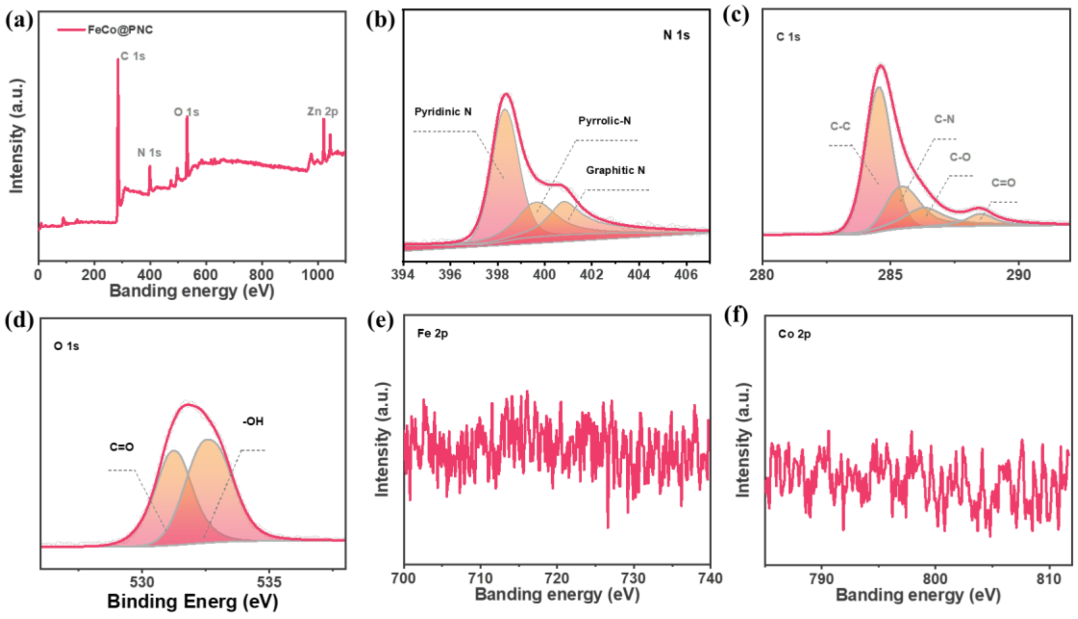

Figure S3. XPS of FeCo@NPC.

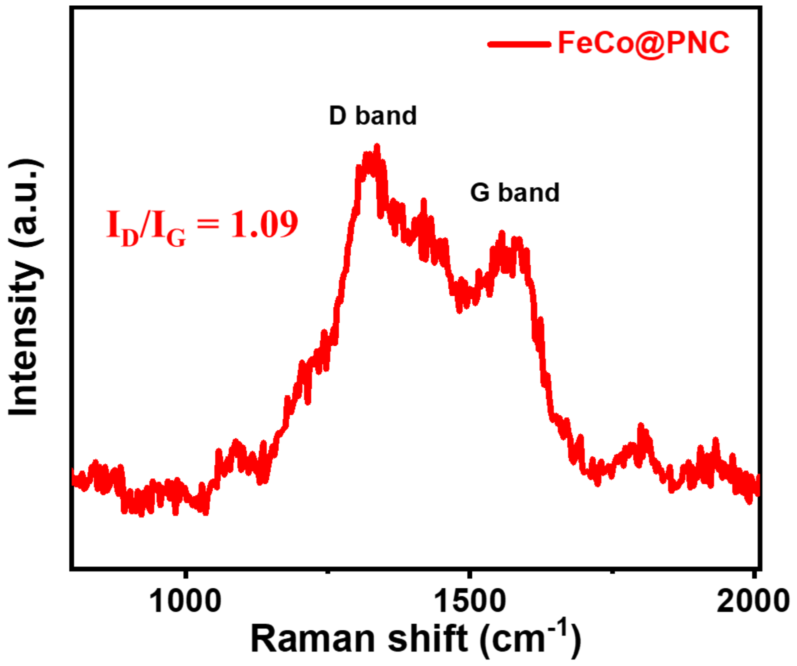

Figure S4. Raman spectra of FeCo@NPC.

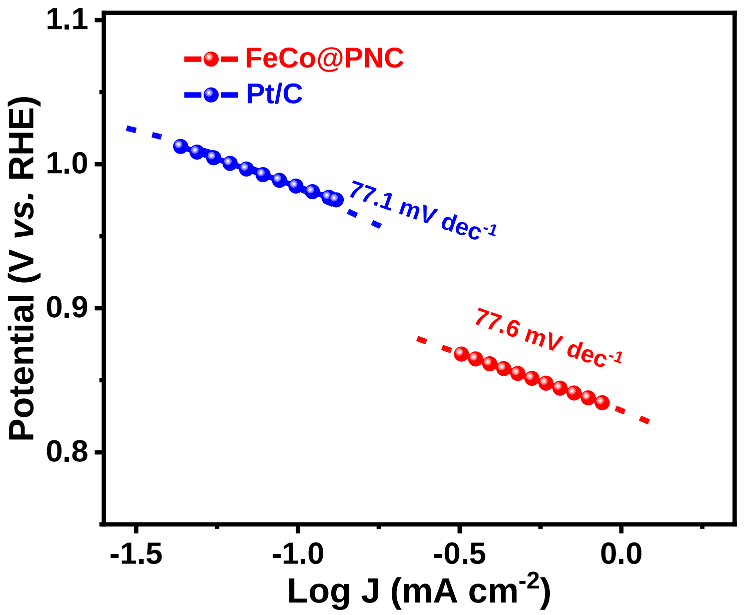

Figure S5 Tafel curves of FeCo@NPC and Pt/C toward ORR.

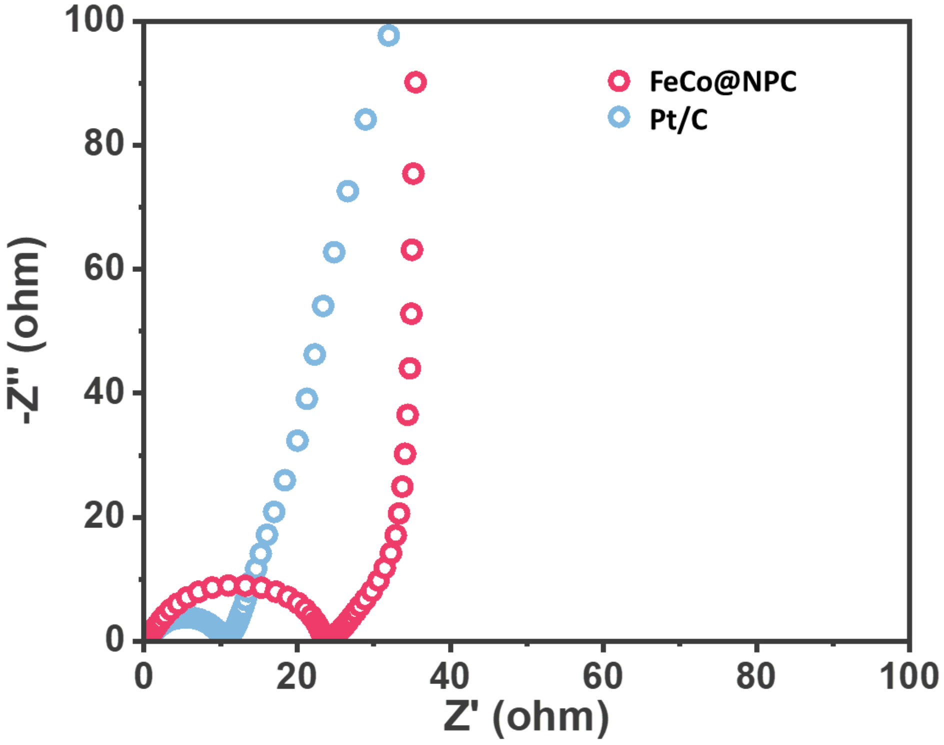

Figure S6 Nyquist plots of FeCo@NPC and Pt/C.

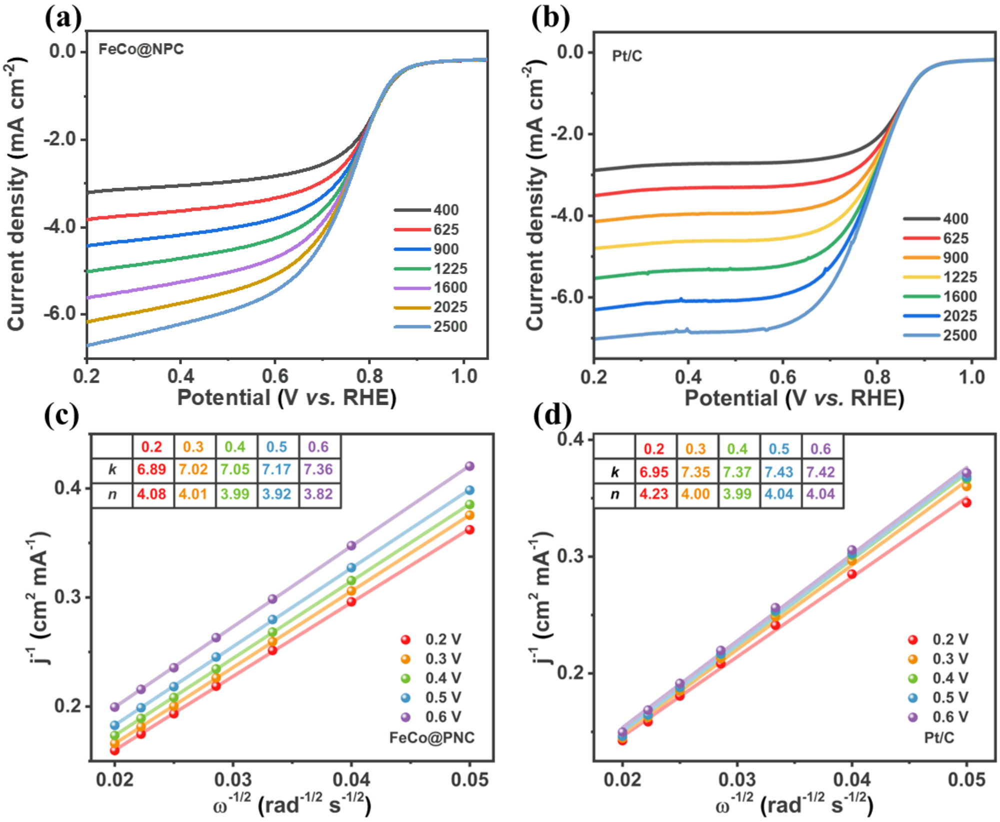

Figure S7. LSV curves at various rotating speed of (a) FeCo@NPC; (b) Pt/C. Corresponding K-L plots at different potentials of (c) FeCo@NPC, and (d) Pt/C.

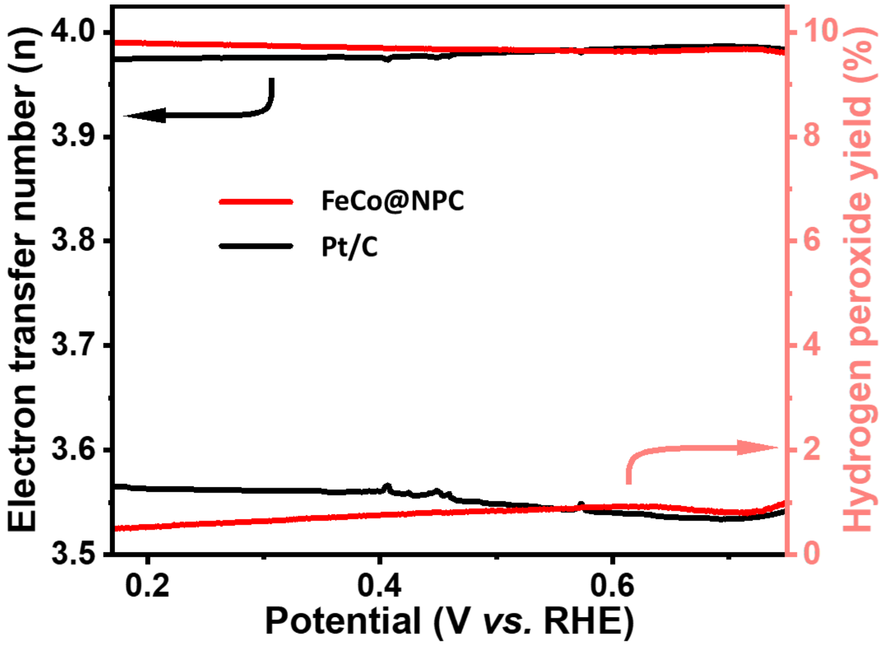

Figure S8. Transfer electron number and H_2O_2 production of FeCo@NPC and Pt/C catalysts, respectively, which were calculated from RRDE measurements.

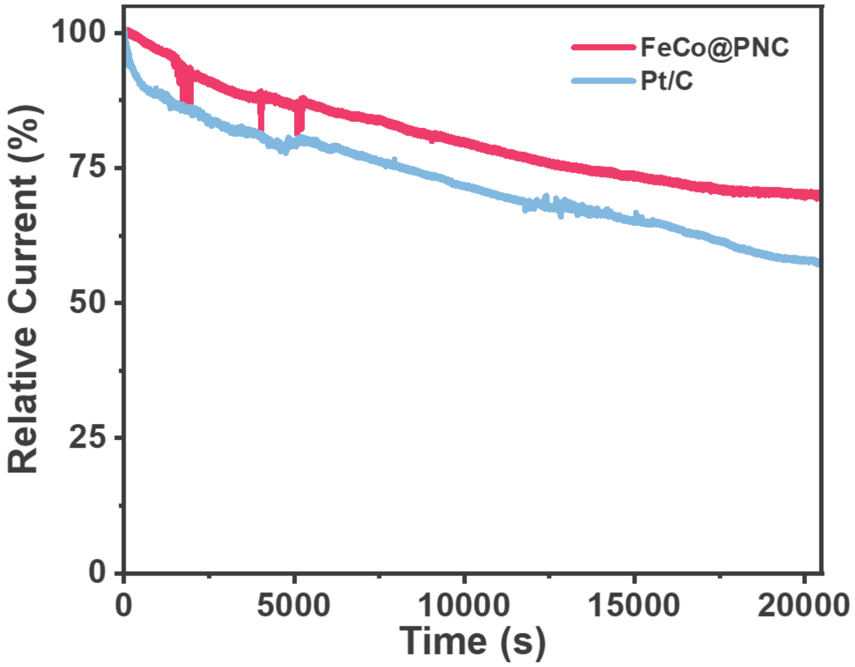

Figure S9. The stability test of FeCo@NPC and Pt/C toward ORR by employing

chronoamperometry.

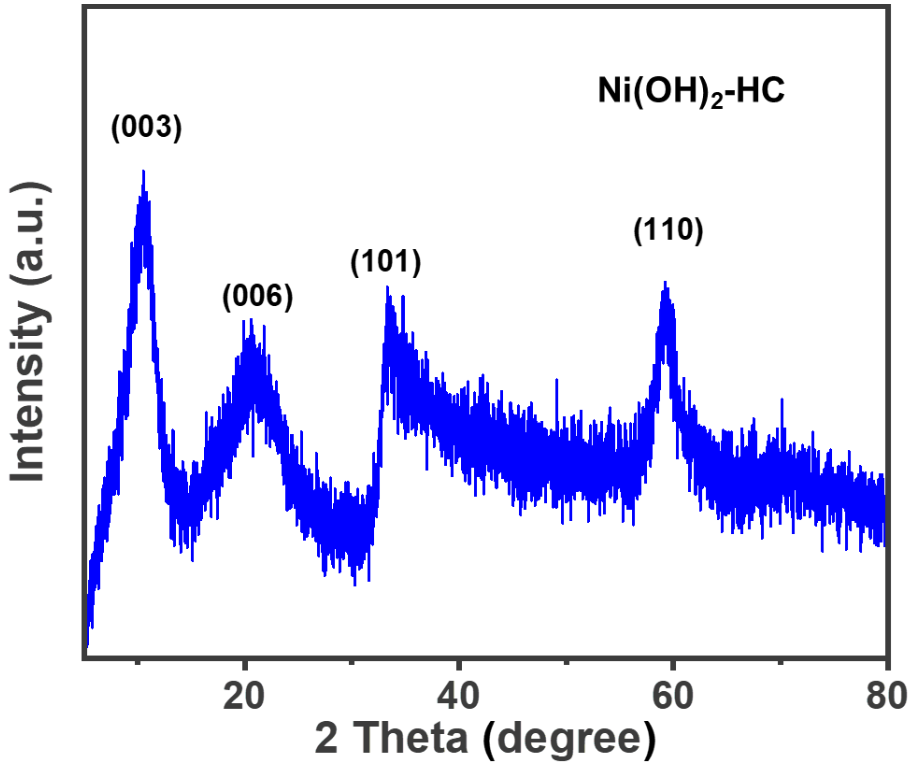

Figure S10. XRD pattern of $Ni(OH)_2$-HC

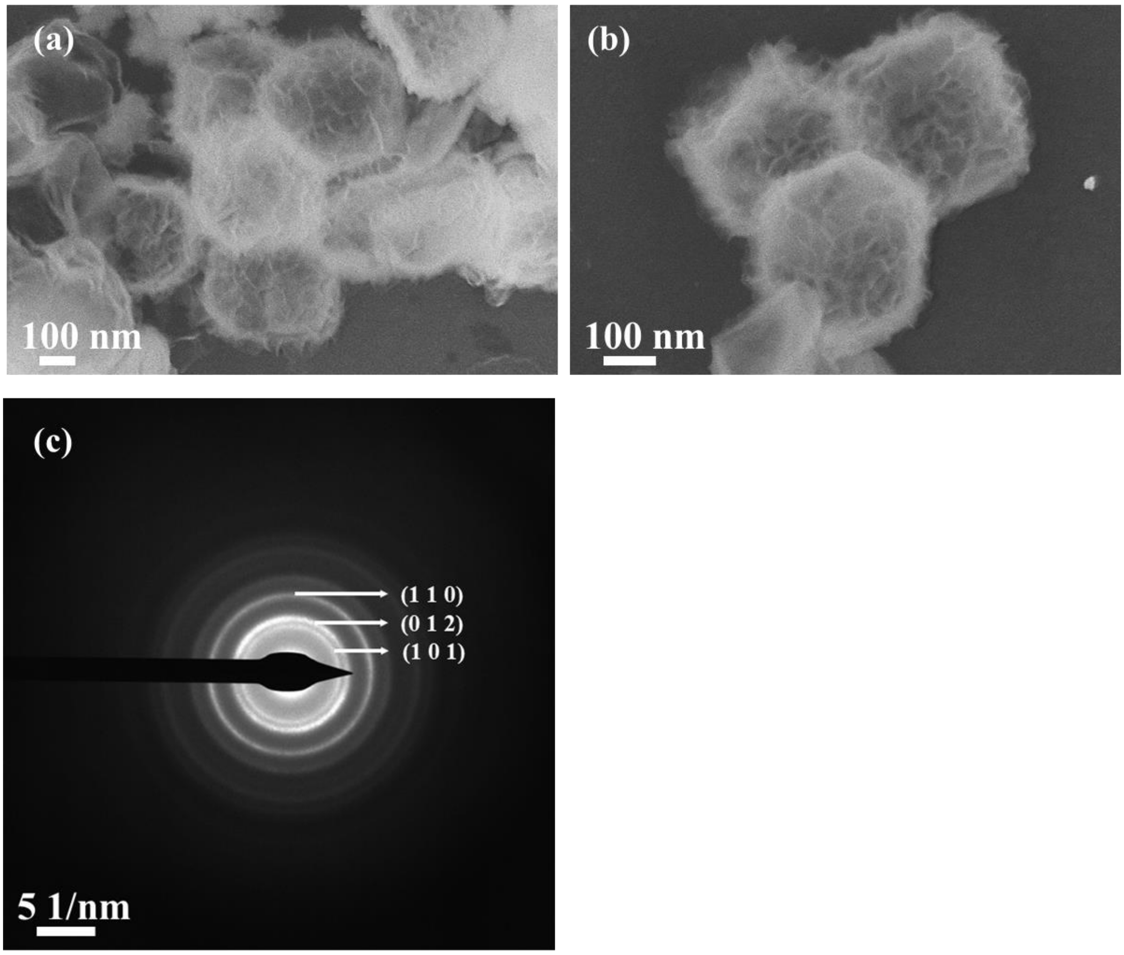

Figure S11. (a, b) SEM images; (c) elected area electron diffraction pattern of $Ni(OH)_2$-HC.

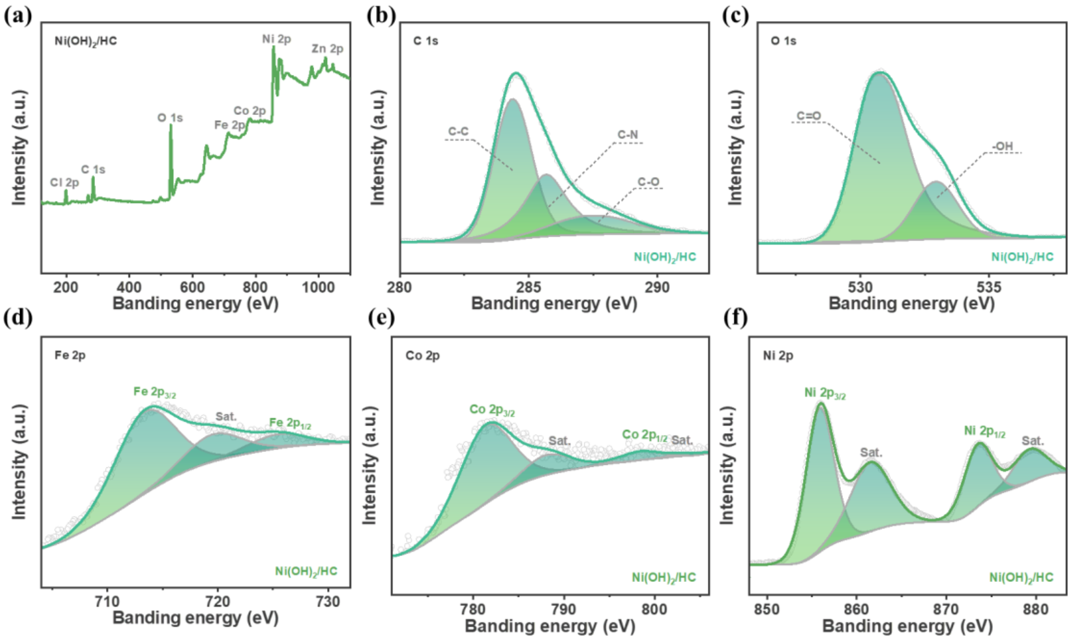

Figure S12. XPS of $Ni(OH)_2$-HC.

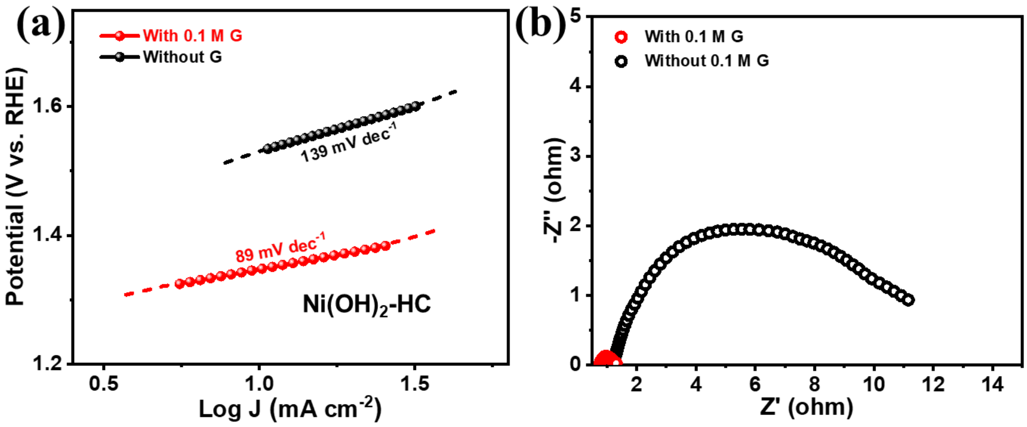

Figure S13. (a) Tafel plots; (b) Nyquist curves of $Ni(OH)_2$-HC with and without glycerin.

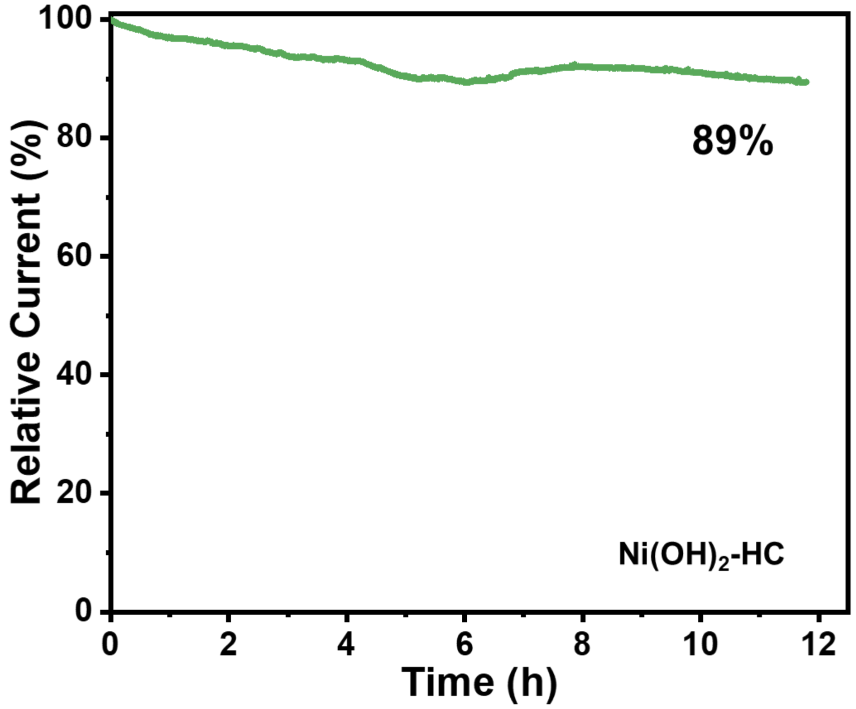

Figure S14. The stability test of $Ni(OH)_2$-OH toward GOR by employing chronoamperometry.

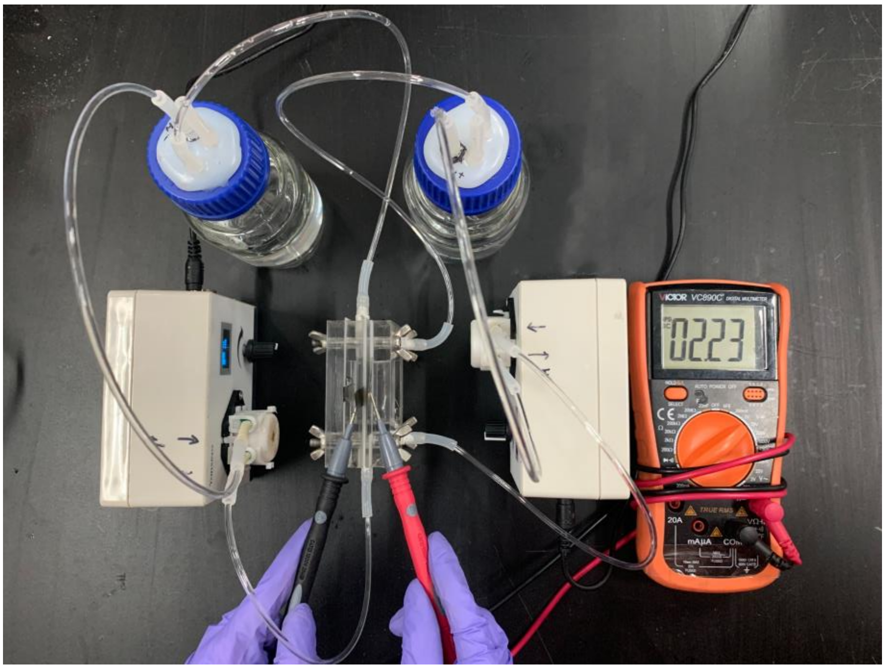

Figure 15. Digital photograph of as-built h-RZAB.

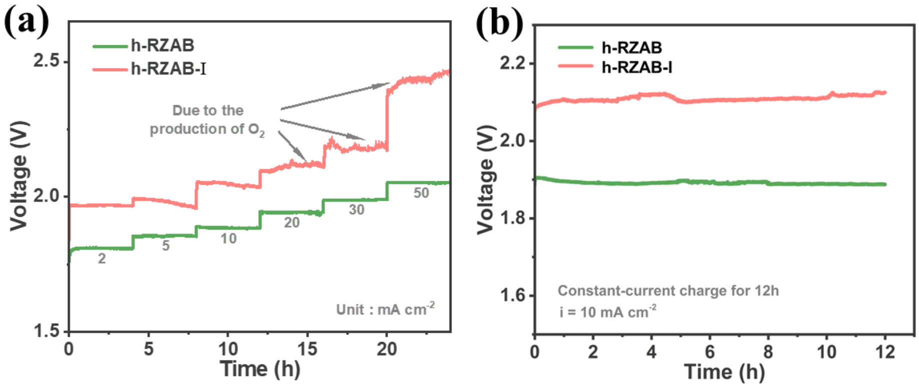

Figure S16 (a) Charging voltage at different current densities; (b) Galvanostatic charging curves at a current density of 10 mA cm^{-2} of h-RZAB and h-RZAB-I.

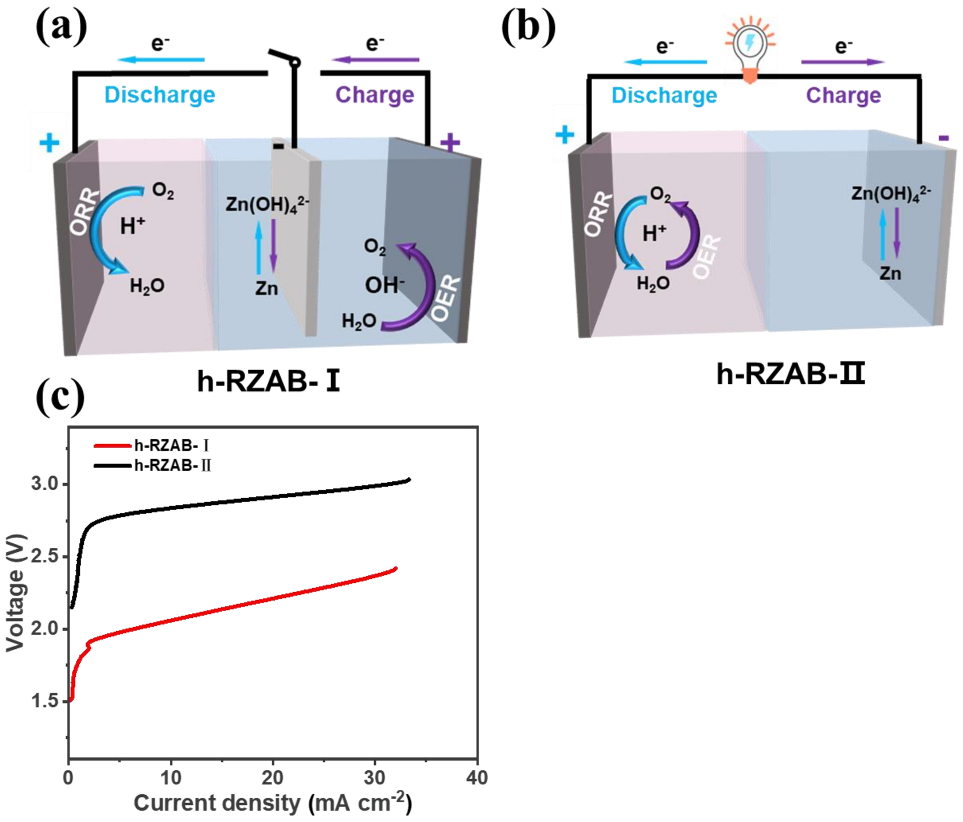

Figure S17. (a, b) Schematic diagram; (c) The charging polarization curves of h-RZAB-I and h-RZAB-II.

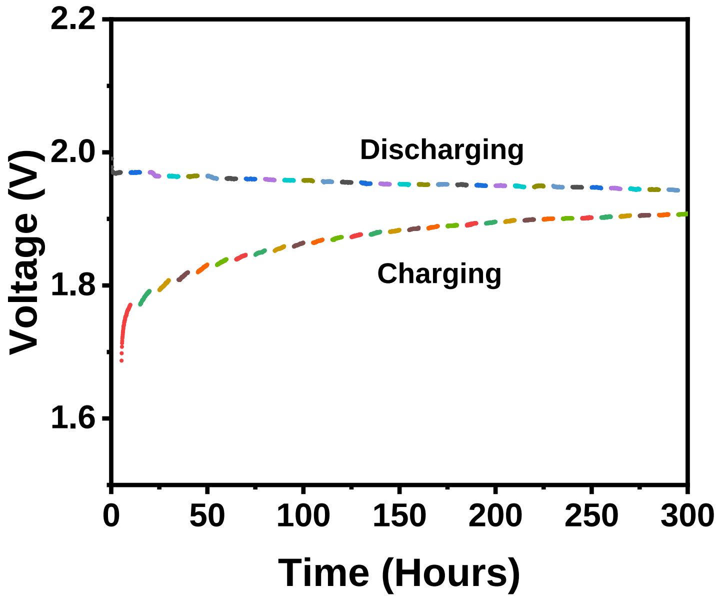

Figure S18. The stability of as-proposed h-RZAB.

Table S1 Comparisons of Zn-air batteries in terms of performance parameters such as

battery, catalysts, open circuit potential, peak power density, and energy density.

**Battery Type**	**Catalysts**	**Open circuit potential (V)**	**Peak power density (mW cm^{-2})**	**Energy density (Wh kg^{-1})**	**Ref.**
Conventional	Co@NPCP/NB-CNF-2-800	1.48	143.8	/	[1]
Conventional	Co@Co-CNTs/PNAs	1.51	371.6	/	[2]
Conventional	FeCo/Se-CNT	1.54	173.4	/	[3]
Conventional	Co@hNCTs-800	1.45	149	/	[4]
Conventional	Co_2P/CoN_4@NSC-500	1.45	134.5	/	[5]
Conventional	FeN_4/S-N-Gra	~ 1.5	578	/	[6]
Hybrid	Commercial Pt/C	2.25	380	1522	[7]
Hybrid	h-NNC-1150	2.11	270	1279	[8]
Hybrid	FeCo@NPC	2.23	562.7	1498	This work

References:

[1] C. Gu, J. Li, J.-P. Liu, H. Wang, Y. Peng, C.-S. Liu, *Appl. Catal. B: Environ.* **2021**, *286*, 119888.

[2] X. Shu, Q. Chen, M. Yang, M. Liu, J. Ma, J. Zhang, *Adv. Energy Mater.* **2023**, *13*, 2202871.

[3] H. Zhang, M. Zhao, H. Liu, S. Shi, Z. Wang, B. Zhang, L. Song, J. Shang, Y. Yang, C. Ma, L. Zheng, Y. Han, W. Huang, *Nano Lett.* **2021**, *21*, 2255-2264.

[4] Q. Zhou, Z. Zhang, J. Cai, B. Liu, Y. Zhang, X. Gong, X. Sui, A. Yu, L. Zhao, Z. Wang, Z. Chen, *Nano Energy* **2020**, *71*, 104592.

[5] Y.-X. Zhao, J.-H. Wen, P. Li, P.-F. Zhang, S.-N. Wang, D.-C. Li, J.-M. Dou, Y.-W. Li, H.-Y. Ma, L. Xu, *Angew. Chem. Int. Ed.* **2023**, *62*, e202216950.

[6] Y. Wang, Y. Zhang, H. Huang, J. Qiao, *ACS Sustain. Chem. Eng.* **2020**, *8*, 731-738.

[7] P. Cai, Y. Li, J. Chen, J. Jia, G. Wang, Z. Wen, *ChemElectroChem* **2018**, *5*, 589-592.

[8] P. Cai, X. Peng, J. Huang, J. Jia, X. Hu, Z. Wen, *Sci. China Chem.* **2019**, *62*, 385-392.
